# Corticostriatal Field Potentials Are Modulated at Delta and Theta Frequencies during Interval-Timing Task in Rodents

**DOI:** 10.3389/fpsyg.2016.00459

**Published:** 2016-04-05

**Authors:** Eric B. Emmons, Rafael N. Ruggiero, Ryan M. Kelley, Krystal L. Parker, Nandakumar S. Narayanan

**Affiliations:** ^1^Department of Neurology, Carver College of Medicine, The University of IowaIowa City, IA, USA; ^2^Department of Neuroscience and Behavioral Sciences, University of São PauloSão Paulo, Brazil; ^3^Aging Mind and Brain Initiative, Carver College of Medicine, The University of IowaIowa City, IA, USA

**Keywords:** prefrontal cortex, striatum, dorsomedial striatum, Parkinson’s disease, medial frontal cortex, local field potential, temporal control, interval timing

## Abstract

Organizing movements in time is a critical and highly conserved feature of mammalian behavior. Temporal control of action requires corticostriatal networks. We investigate these networks in rodents using a two-interval timing task while recording LFPs in medial frontal cortex (MFC) or dorsomedial striatum. Consistent with prior work, we found cue-triggered delta (1–4 Hz) and theta activity (4–8 Hz) primarily in rodent MFC. We observed delta activity across temporal intervals in MFC and dorsomedial striatum. Rewarded responses were associated with increased delta activity in MFC. Activity in theta bands in MFC and delta bands in the striatum was linked with the timing of responses. These data suggest both delta and theta activity in frontostriatal networks are modulated during interval timing and that activity in these bands may be involved in the temporal control of action.

## Introduction

The cortex and striatum are critical for the temporal control of action in mammals (Buhusi and Meck, 2005). These regions are dysfunctional in neuropsychiatric disorders such as schizophrenia and PD, resulting in impaired temporal processing and other cognitive deficits (Malapani et al., 1998; Matell et al., 2003; Ward et al., 2012; Parker et al., 2015). The underlying mechanisms of temporal control by corticostriatal systems remain unclear. A better understanding of these circuits could provide insight into both mammalian behavior and human disease.

Temporal control of action can be studied using an interval-timing task. This task requires subjects to estimate an interval of several seconds by making a motor response. Interval timing requires both working memory for temporal rules and attention to the passage of time. Goal-directed timing behavior also shares resources with other executive processes (Brown et al., 2013; Parker et al., 2013). In both humans and rodents, prefrontal areas and dorsal striatum are required for temporal processing (Meck and Benson, 2002; Meck, 2006; Coull et al., 2011). In rodents, inactivation of medial frontal cortex (MFC) impairs interval timing (Uylings et al., 2003; Narayanan et al., 2012; Kim et al., 2013). MFC projects to dorsal and medial regions of the rodent striatum which are also required for temporal control of action, unlike the ventral striatum (Matell and Meck, 2004; Meck, 2006; Kurti and Matell, 2011).

In medial frontal regions of humans and rodents, low-frequency activity is associated with cognitive control and organizing goal-directed activity in time (Cavanagh et al., 2012; Narayanan et al., 2013; Cavanagh and Frank, 2014). Activity around 4 Hz coordinates task information in prefrontal, midbrain, and hippocampal areas (Fujisawa and Buzsáki, 2011). However, it is unclear if activity in this band extends to the basal ganglia. Strikingly, cue-triggered activity in delta and theta bands in MFC is highly conserved in humans and rodents during timing tasks (Narayanan et al., 2013; Parker et al., 2015). In frontal cortex, activity in these bands is coherent with neurons that may encode the accumulation of temporal information (Parker et al., 2014). In both humans and rodents, this cue-triggered delta and theta activity depends on dopamine via medial frontal D1 dopamine receptors (Parker et al., 2015). In the striatum, the striatal beat frequency model proposes that oscillations in the activity of individual neurons may act as a mechanism for the representation of time (Matell and Meck, 2004; Oprisan and Buhusi, 2011). These and many other findings suggest that low-frequency activity may be an important component of temporal processing (Gu et al., 2015).

To further explore the role of low-frequency activity in temporal processing, we recorded LFPs from rodent MFC and the dorsomedial striatum during performance of an interval-timing task with two intervals in rodents. Because our prior work has found delta (1–4 Hz) and theta (4–8 Hz) activity associated with temporal processing, we restricted our analyses to these bands in the present manuscript (Narayanan et al., 2013; Cavanagh and Frank, 2014; Parker et al., 2014, 2015; Laubach et al., 2015). We tested the hypothesis that delta/theta activity is related to temporal processing in corticostriatal circuits. We found cue-triggered activity in delta and theta bands in MFC. Delta activity was found in MFC and striatum across temporal intervals, and was observed around rewarded responses in MFC. Frontostriatal delta/theta activity was related to when animals responded in time during the interval. These data indicate that delta/theta activity in corticostriatal circuits is involved in the temporal control of action.

## Materials and Methods

### Subjects

Eight Long-Evans rats (age 2 months; 200–225 g) were trained to perform an interval-timing task using standard operant procedures. Animals were motivated by regulated access to water, while food was available *ad libitum*. Rats consumed 5–6 ml of water/100 g body weight each day. 5–10 ml were consumed during the behavioral session and any additional water needed was provided 1–3 h after each behavioral session in the home cage. Rats were singly housed and kept on a 12-h light/dark cycle; all experiments took place during the light cycle. Rats were kept at ∼90% of their free-access body weight during these experiments, and received 1 day of free access to water per week. All procedures were approved by the Animal Care and Use Committee at the University of Iowa.

### Interval-Timing Task

**FIGURE 1:**
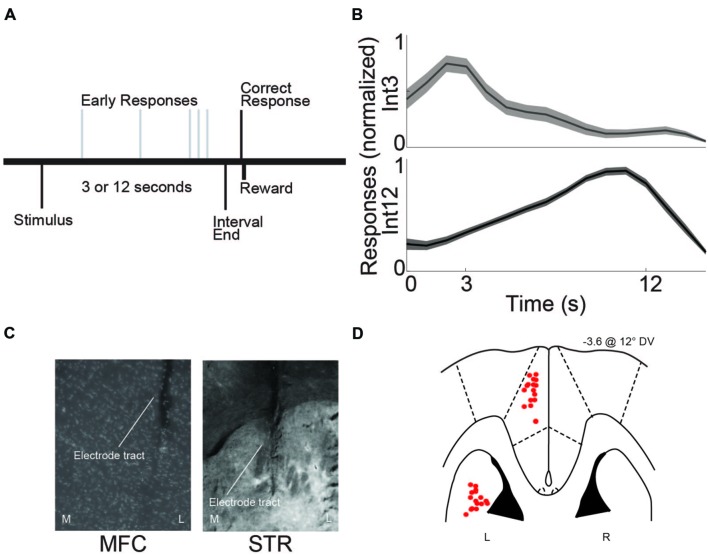
**Interval-timing task and histological validation. (A)** Rats estimated a 3 or 12 s interval by pressing a lever. The first response after the end of the interval was rewarded with water. Multiple responses per trial were permitted. **(B)** Average response-timing curves from all eight animals included in the study. Responses were normalized within both 3 s (Int3) and 12 s (Int12) trials. **(C)** Representative photomicrograph of electrode tracts in the left hemisphere of medial frontal cortex (MFC) and dorsomedial striatum (STR) from a coronal view. M, Medial, L, Lateral. **(D)** Horizontal image of histological reconstruction from the four animals implanted with multielectrode arrays either in the MFC or STR. L, Left, R, Right. Red circles correspond to the locations of electrodes used in the study.

Rats were trained on the interval-timing task with a standard operant approach described in detail previously (Narayanan et al., 2012; Parker et al., 2013). First, animals went through fixed-ratio training to make operant lever presses to receive water reward. Next, animals were trained in a 12 s fixed-interval timing task where rewards were delivered for responses made following a 12 s interval (**Figure 1A**). Rewarded presses were signaled by a click and an ‘off’ houselight. Each rewarded trial was immediately followed by a 6, 8, 10, or 12 s pseudorandom intertrial interval which concluded with an ‘on’ houselight signaling the beginning of the next trial. Responses occurring before 12 s were not reinforced. The houselight stayed on from trial onset until the onset of the intertrial interval. Training sessions were 60 min long. Importantly, rodents were allowed to make multiple responses per trial. Average response times were used to determine central tendency of response time per trial. The timing of each response was used to generate time-response histograms. To compare across animals, time-response histograms were normalized to the highest response rate during the interval. After animals learned the 12 s interval—indicated by a peak in their time-response histograms around 12 s—a second delay of 3 s was added. This 3 s interval was signaled with an additional light on the right side of the lever. Operant chambers (MedAssociates, St Albans, VT, USA) were equipped with a lever, a drinking tube, and a speaker driven to produce an 8 kHz tone at 72 dB. Behavioral arenas were housed in sound-attenuating chambers (MedAssociates). Water rewards were delivered via a pump (MedAssociates) connected to a standard metal drinking tube (AnCare) via Tygon tubing.

### Surgical and Perfusion Procedures

Rats trained in the interval-timing task were implanted with a microwire array in MFC or dorsomedial striatum according to procedures described previously (Narayanan and Laubach, 2006). Briefly, animals were anesthetized using Ketamine (100 mg/kg) and Xylazine (10 mg/kg). A surgical level of anesthesia was maintained with hourly (or as needed) Ketamine supplements (10 mg/kg). Under aseptic surgical conditions, the scalp was retracted and the skull was leveled between bregma and lambda. A craniotomy was drilled over the area above MFC or dorsomedial striatum and four holes were drilled for skull screws. A microelectrode array consisting of 50 μm stainless steel wires (250 μm between wires and rows; impedance measured *in vitro* at ∼400 kΩ; Plexon: Dallas, TX) configured in 4 × 4 (*n* = 4) or 2 × 8 (*n* = 4) was implanted in eight animals in either MFC (coordinates from bregma: AP +3.2, ML ± 1.2, DV -3.6 @ 12° in the lateral plane) or in dorsomedial striatum (coordinates from bregma: AP +0.0, ML ± 4.2, DV -3.6 @ 12° in the lateral plane). The electrode ground wire was wrapped around the skull screws. Electrode arrays were inserted while recording neuronal activity to verify implantation in layer II/III of MFC or in the most dorsal portion of dorsomedial striatum. The craniotomy was sealed with cyanoacrylate (‘SloZap’, Pacer Technologies, Rancho Cucamonga, CA, USA) accelerated by ‘ZipKicker’ (Pacer Technologies) and methyl methacrylate (i.e., dental cement; AM Systems, Port Angeles, WA, USA). Following implantation, animals recovered for 1 week before being reacclimatized to behavioral and recording procedures.

Following experiments, rats were anesthetized, sacrificed by injections of 100 mg/kg sodium pentobarbital, and transcardially perfused with 4% formalin. Brains were post-fixed in a solution of 4% formalin and 20% sucrose before being sectioned on a freezing microtome. Brain slices were mounted on gelatin-subbed slides and stained for cell bodies using DAPI. Histological reconstruction was completed using postmortem analysis of electrode placements by confocal microscopy or stereology microscopy in each animal. These data were used to determine electrode location within MFC or dorsomedial striatum (**Figures 1C,D**).

### Neurophysiological Recordings

Neuronal ensemble recordings in MFC or dorsomedial striatum were made using a multi-electrode recording system (Plexon, Dallas, TX, USA). LFPs were recorded using wide-band boards with bandpass filters between 0.07 and 8000 Hz. Analysis of neuronal activity and quantitative analysis of basic firing properties were carried out using NeuroExplorer (Nex Technologies, Littleton, MA, USA) and with custom routines for MATLAB. Microwire electrode arrays were comprised of 16 electrodes. In each animal, one electrode without single units was reserved for local referencing and filtering out of noise, yielding 15 electrodes per rat. LFPs were recorded from four low-noise electrodes in each rodent. We recorded LFPs using wide-band boards with analog filters between 0.7 and 100 Hz.

### Time-Frequency Analyses

In line with our previous work, all analyses were restricted to delta and theta bands (Narayanan et al., 2013; Cavanagh and Frank, 2014; Parker et al., 2014, 2015; Laubach et al., 2015). Time-frequency calculations were computed using custom-written MATLAB routines (Cavanagh et al., 2009). Time-frequency measures were computed by taking the inverse FFT of the convolution of a fast Fourier transformed (FFT) LFP power spectrum and a set of complex Morlet wavelets (defined as a Gaussian-windowed complex sine wave: $e^{i\,2\,\pi \,t\,f}\,e^{-\frac{t^{2}}{2\times \sigma^{2}}}$ where *t* is time, *f* is frequency [increasing from 1 to 50 Hz in 50 logarithmically spaced steps], and σ is scaling, defined as cycles/(2πf), with four cycle wavelets) (Narayanan et al., 2013; Parker et al., 2014, 2015; Laubach et al., 2015). We varied the number of cycles and other parameters to balance time-frequency resolution for the bands we were interested in here (delta/theta bands) and the time windows used for analysis (∼1 s). Wavelet transformation results in estimates of instantaneous power which were subsequently normalized to a decibel (dB) scale (*10^∗^log10[power(t)/power(baseline)]*), allowing a direct comparison of effects across frequency bands. Hypothesis-driven statistical significance was computed via a paired *t*-test in the delta (1–4 Hz) or theta (4–8 Hz) frequency bands by calculating the average power change in a period of interest vs. baseline across all subjects. We defined the baseline period as -500 to -300 ms prior to stimulus presentation. For cue-evoked analyses, 0–500 ms post-stimulus onset was compared to baseline. For whole-trial analyses, 0.5 (to exclude the immediate post-stimulus period) to 3 s post-stimulus onset was compared to baseline for 3 s trials (Int3 trials) and 0.5–12 s post-stimulus onset was compared to baseline for 12 s trials (Int12 trials). For response analyses, mean activity from -500 to 0 ms pre-response in Int3 and Int12 trials was compared to the mean baseline. Rewarded presses were the first press after interval end that resulted in reward (3 s after the cue for Int3; 12 s after the cue for Int12). Unrewarded presses occurred prior to the end of the interval (0–2.9 s for Int3; 0–11.9 s for Int12). To match variance with rewarded trials we randomly subsampled the number of unrewarded presses so that comparisons between rewarded and unrewarded trials had the same number of trials in each category. Error bars were computed from variance across subjects and represent the standard error of the mean.

### Linear Models

To investigate the relationship between the timing of responses and frontostriatal field potentials, we used linear regression (*fitlm.m* in MATLAB) where delta or theta activity calculated from -500–0 ms prior to the response was regressed against the timing of the response. To reduce the role of cue-related activity, this analysis was calculated from 500 ms to 3 s for Int3 trials and 500 ms–12 s for Int12 trials. Delta and theta power were derived according to methods above. Slope was calculated as the change in delta/theta power (ΔdB) over the change in the timing of responses (in seconds). Significant linear fits were derived from analysis of variance.

## Results

### Interval-Timing Behavior

The eight rats used in this study were trained on the 3 and 12 s interval-timing task described above (Int3 and Int12, respectively; **Figure 1A**). The mean response time for Int3 was 4.8 ± 0.28 s and the mean response time for Int12 was 11.7 ± 0.43 s (**Figure 1B**). Mean response times were significantly different on Int3 vs. Int12 trials [*t*_(7)_ = 12.5, *p* < 0.001]. The variability of interval-timing behavior was similar to that seen in previous studies (Kim et al., 2013; Parker et al., 2014, 2015; Xu et al., 2014).

### Cue-Triggered Delta/Theta Activity in Medial Frontal Cortex and Dorsomedial Striatum

**FIGURE 2:**
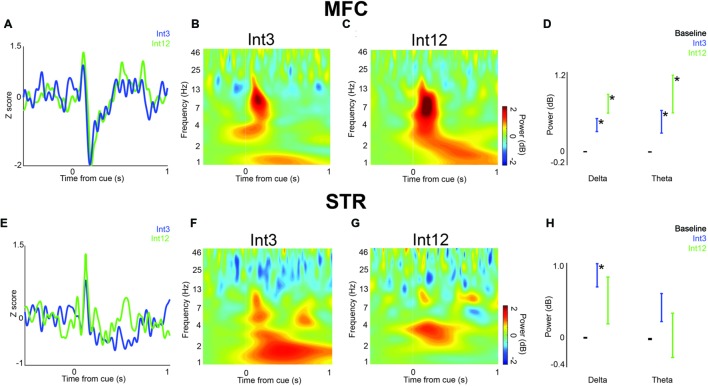
**Cue-related low-frequency activity in MFC and dorsomedial striatum (STR). (A)** LFPs recorded from MFC in four rats showed a cue-evoked ERP following the Int3 cue (blue) and Int12 cue (green). **(B)** Time-frequency analysis of LFPs revealed a delta/theta (3–12 Hz) burst triggered by the Int3 stimulus when the animal responded and was rewarded. **(C)** A delta/theta burst was visible after the Int12 stimulus on rewarded trials. **(D)** There was a significant increase in power in Int3 (blue) and Int12 (green) trials over their respective baselines (black) in the delta (left) and theta (right) frequency bands. Error bars denote variance across subjects. **(E)** LFPs recorded from STR in four rats showed a subtle ERP on Int3 reward trials (blue) and Int12 reward trials (green). Cue-related delta/theta activity in STR on Int3 **(F)** and Int12 **(G)** trials. **(H)** There was a significant increase from baseline at the delta frequency band but not at the theta band on Int3 reward trials (blue) and not at either frequency on Int12 reward trials (green). Error bars denote variance across subjects; Asterisk indicates *p* < 0.05.

To test the idea that delta and theta bands are modulated during interval timing, we recorded field potentials from MFC or dorsomedial striatum of rats trained to perform an interval-timing task (**Figures 1C,D**). In MFC, a large cue-triggered ERP was found (**Figure 2A**). The average latency to the positive peak on Int3 trials was 126 ± 6.4 ms, followed by a negative peak at 208 ± 8.3 ms. On Int12 trials the average latency to the positive peak was 122 ± 5.6 ms, followed by a negative peak at 192 ± 8.4 ms. Time-frequency analysis demonstrated strong delta and theta activity 0–0.5 s after cue onset (**Figures 2B,C**). Direct comparison of MFC activity revealed significant cue-related modulation of delta and theta bands relative to baseline in both Int3 trials [delta: *t*_(15)_ = 4.1, *p* < 0.01; theta: *t*_(15)_ = 2.7, *p* < 0.05] and Int12 [delta: *t*_(15)_ = 5.2, *p* < 0.01; theta: *t*_(15)_ = 3.1, *p* < 0.01; **Figure 2D**]. Notably, cue-related activity was similar on Int3 and Int12 trials (**Figure 2D**). These data indicate that delta and theta modulations early in the trial are cue-triggered and do not significantly differ based on interval length.

In dorsomedial striatum, a less distinct pattern was observed. On Int3 trials, the average latency to the positive peak was 130 ± 4.6 ms, followed by a less prominent negative peak at 192 ± 9.8 ms. On Int12 trials, the average latency to the positive peak was 130 ± 4.2 ms, followed by a less distinct negative potential at 178 ± 10.9 ms. Low-frequency modulation by the cue was visible on both trial types (**Figures 2E–G**). However, there was only a significant increase from baseline in the striatal delta band on Int3 trials [delta: *t*_(15)_ = 4.7, *p* < 0.01; **Figure 2H**]. Taken together, these data demonstrate that cue-related delta and theta activity is primarily modulated in MFC.

### Interval-Related Delta/Theta Activity in Medial Frontal Cortex and Dorsomedial Striatum

**FIGURE 3:**
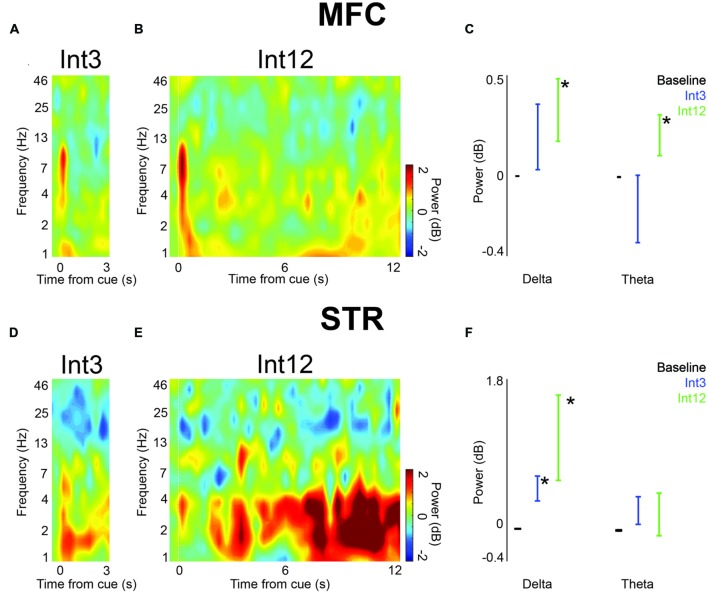
**Low-frequency activity power increased in trials with reward in MFC and dorsomedial striatum (STR) across the entire interval. (A)** On Int3 trials, time-frequency analysis showed cue-related delta/theta activity and elevated delta activity throughout the interval. **(B)** Time-frequency analysis in MFC revealed ∼4-Hz activity over Int12 trials. **(C)** A significant increase in power over their respective baselines was visible in the delta and theta frequency bands on Int12 reward trials. Error bars denote variance across subjects. **(D)** Delta/theta activity was visible in STR on Int3 and **(E)** Int12 trials. **(F)** Significantly greater power was visible on Int3 and Int12 trials in the delta band in STR. Theta power was not significantly higher than baseline on either trial type. Error bars denote variance across subjects. Asterisk indicates *p* < 0.05.

Next, we examined field potentials over the duration of the trial in MFC and dorsomedial striatum. To compare Int3 and Int12 trials, we averaged activity in delta and theta bands across the interval. In MFC, we found that average delta and theta activity bands across the interval were significantly higher than baseline for Int12 trials [0.5–12 s following cue; delta: *t*_(15)_ = 2.4, *p* < 0.05; theta: *t*_(15)_ = 2.4, *p* < 0.05; **Figures 3A–C**]. No significant difference from baseline was seen on Int3 trials. These data indicate that medial frontal delta activity is engaged on longer intervals.

In the dorsomedial striatum, only delta activity was significantly higher than baseline for Int3 and Int12 conditions [Int3—delta: *t*_(15)_ = 3.2, *p* < 0.01; Int12—delta: *t*_(15)_ = 2.5, *p* < 0.05; **Figures 3D–F**]. These data suggest that delta activity is modulated across temporal intervals in frontostriatal circuits.

### Medial Frontal Delta Activity Is Related to Rewarded Responses

**FIGURE 4:**
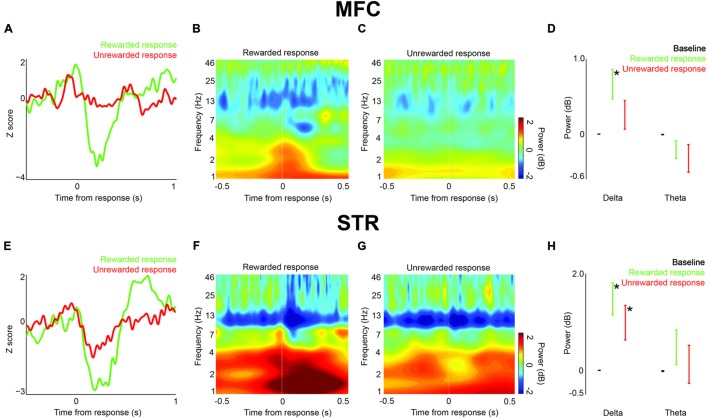
**Local field potential response-related activity in MFC and dorsomedial striatum (STR) during interval timing. (A)** An ERP was visible in MFC on rewarded (green) vs. unrewarded (red) responses. **(B)** Time-frequency analysis around press events showed an increase in delta/theta power around rewarded press events. **(C)** Relatively low power was visible around unrewarded presses. **(D)** There was a significant increase in delta power prior to rewarded presses over both baseline and delta power prior to unrewarded presses. Error bars denote variance across subjects. **(E)** ERPs in STR both with rewarded (green) and unrewarded (red) presses. **(F)** An increase in delta power was visible prior to and especially after rewarded presses. **(G)** An increase in delta/theta power was also visible around unrewarded presses. **(H)** There was a significant increase in delta power over baseline prior to both rewarded and unrewarded presses. Error bars denote variance across subjects. Asterisk indicates *p* < 0.05.

Next, we analyzed field potentials around lever presses. Trials on which the animal pressed the lever after the 12 s interval were rewarded. We found marked press-related potentials in both MFC and dorsomedial striatum (**Figure 4A**). The average latency to the positive peak in MFC on rewarded trials was -8 ± 16.5 ms, followed by a negative peak at 206 ± 11.6 ms. The average latency to the positive peak on unrewarded responses was -82 ± 8.5 ms, followed by a negative peak at 6 ± 10.9 ms. In MFC, only delta activity was significantly higher on rewarded responses both compared to baseline [*t*_(15)_ = 3.0, *p* < 0.05] and compared to unrewarded presses [*t*_(15)_ = 2.6, *p* < 0.05; **Figures 4B–D**].

A similar press-related potential was found in dorsomedial striatum (**Figure 4E**). In dorsomedial striatum, the average latency to the positive peak on rewarded responses was 4 ± 11.8 ms, followed by a negative peak at 190 ± 18.3 ms. The average latency to the positive peak on unrewarded responses was -44 ± 10.4 ms, followed by a negative peak at 172 ± 18.0 ms. In contrast to MFC, striatal delta power was significantly higher than baseline on both rewarded and unrewarded responses [rewarded: *t*_(15)_ = 4.3, *p* < 0.01; unrewarded: *t*_(15)_ = 2.6, *p* < 0.05; **Figures 4B–H**]. There was not a significant difference between rewarded and unrewarded responses in either the delta or theta bands. Thus, in MFC delta activity was associated with rewarded presses, while in dorsomedial striatum delta activity was associated with all lever presses. These data provide insight into delta and theta activity throughout corticostriatal circuits during interval timing.

### Delta/Theta Activity and Temporal Control of Responding

To examine how delta/theta activity in the MFC and striatum predicted when animals responded, we used linear models of frontostriatal field potential activity vs. response time. We examined delta and theta activity -500–0 ms prior to lever press. Significant linear fits are indicated in **Table 1** as changes in delta or theta power in dB per second of response time. In MFC, theta activity immediately prior to lever press predicted when animals responded for both Int3 and Int12 trials (Int3: *p* < 0.03; Int12: *p* < 0.02). By contrast, in dorsomedial striatum delta activity immediately prior to lever press predicted when animals responded (Int3: *p* < 0.0001; Int12: *p* < 10^-8^). These data indicate that response-related theta activity in MFC and delta activity in the striatum depends on when animals press the lever during the interval, and indicate that these bands are involved in the temporal control of action in frontostriatal circuits.

**Table 1:** Relationship of response-related delta/theta power compared to the timing of responses by interval length (Int3–3 s intervals; Int12–12 s intervals): Output of the linear regression model calculated for the change in power (ΔdB) over time (s).

		DELTA		THETA	
		Slope (ΔdB/s)	*p*-value	Slope (ΔdB/s)	*p*-value
Int3	MFC	0.20	0.21	-0.38	0.03
	STR	1.77	0.0001	-0.11	0.76
Int12	MFC	0.01	0.50	0.05	0.02
	STR	0.36	10^-8^	0.17	10^-6^

*Significant linear fits were found for theta activity in the medial frontal cortex (MFC) and delta activity in the striatum (STR) in relation to the timing of response (ΔdB/s)*.

## Discussion

Here we studied rodent frontostriatal circuits using LFPs during an interval-timing task. Because our previous work implicates low-frequency activity in delta and theta ranges in the temporal control of action, we focused on these bands in this study. We report four main findings. First, we observed cue-triggered modulations in delta and theta activity primarily in MFC. Second, we found delta activity in MFC and dorsomedial striatum across the temporal interval. Thirdly, we observed increased delta activity in MFC prior to rewarded responses, while striatal delta modulation was observed prior to all responses. Finally, theta activity in MFC and delta activity in the striatum was related to when animals responded during the interval. These data contribute to an understanding of low-frequency activity in corticostriatal circuits that is highly conserved across humans and rodents (Cavanagh et al., 2012; Narayanan et al., 2013; Parker et al., 2015). This similarity could help approach human EEG as well as human intracortical recordings from patients undergoing epilepsy or deep-brain stimulation surgeries (Brown and Williams, 2005; Emeric et al., 2008; Kingyon et al., 2015).

The low-frequency activity observed in MFC after the instructional cue is broadly consistent with past research. Frontal theta and delta bands during elementary cognitive tasks are similar between humans and rodents (Narayanan et al., 2013; Parker et al., 2015; Warren et al., 2015). To our knowledge these are the first field potential data from the dorsomedial striatum in rodents during a timing task. Delta and theta bands have been associated with errors, conflict, working memory, and attention (Curtis and D’Esposito, 2003; Emeric et al., 2008; Liebe et al., 2012; Totah et al., 2013; Cavanagh and Frank, 2014; Chen et al., 2014; Parker et al., 2014; Laubach et al., 2015). Activity in this range may provide a means of synchronizing frontal activity with other brain regions (Fujisawa and Buzsáki, 2011). The pronounced burst of low-frequency activity in MFC following the cue is similar to that seen in our previous work (Parker et al., 2014, 2015). This activity was not unique to either one of the interval lengths—it is likely related to the salience of the cue and communicates the need for cognitive control (Cavanagh and Frank, 2014).

Low-frequency activity was observed throughout the duration of interval-timing tasks in MFC and dorsomedial striatum. Delta and theta activity was significantly increased in MFC on longer-interval trials and was increased on both trial types in dorsomedial striatum. This result suggests that sustained low-frequency activity in the MFC is more engaged on intervals of longer, more demanding duration. Moreover, low-frequency activity in both areas was significantly related to when animals made a response in time. That is, activity in these bands was different if the animal pressed the lever early or late in the interval, indicating that pre-response delta/theta activity can be influenced by temporal preparation of responding (**Table 1**).

Strong reward-related delta activity was observed around responses. Delta activity was increased in dorsomedial striatum on all responses, regardless of reward. Delta activity has been reported from rodent cortex and striatum, and has been associated with motor action, reward processing, and temporal expectation (Stefanics et al., 2010; Cavanagh et al., 2012; Laubach et al., 2015). We observed different relationships between delta activity and interval-timing behavior in the MFC and dorsomedial striatum. One possibility is that medial frontal delta activity reflects reward anticipation during interval timing (Cavanagh et al., 2012; Narayanan et al., 2013; Parker et al., 2014, 2015).

Low frequencies in MFC may represent temporal processing while field potentials in dorsomedial striatum may also reflect the motor output of this processing. Many lines of evidence suggest that the striatum is critical for interval timing (Matell et al., 2003; Matell and Meck, 2004; Meck, 2006; Coull et al., 2011; Merchant et al., 2013). Notably, spiking activity in striatal ensembles robustly encodes temporal processing (Matell et al., 2003; Mello et al., 2015). In contrast, LFP may reflect input to the striatum from a variety of sources (Wall et al., 2013)—MFC being but one of them—making temporal signals relatively difficult to isolate at the level of field potentials. High-frequency gamma and beta activity in the primate striatum have been linked to interval timing, particularly in terms of coherence and entrainment of neural populations (Bartolo et al., 2014). It remains to be seen how striatal field potentials couple with neuronal activity in other brain areas such as MFC.

This study is limited by several factors. Rodent LFP recordings are not a perfect analog to EEG in human subjects, though progress has been made recently in comparing these two systems (Narayanan et al., 2013; Parker et al., 2015; Warren et al., 2015). Due to the scope of this study, we constrained our analyses to delta and theta activity as we have found these bands to be reliably modulated in prior human and rodent work during timing tasks (Narayanan et al., 2013; Parker et al., 2014, 2015; Laubach et al., 2015). Although striatal delta power was distinct on unrewarded vs. rewarded lever presses and correlated with response time, rewarded presses generally occur when the response rate is high and could be affected by movement. By contrast, in MFC, theta power had a more complex relationship with movement on Int3 and Int12 trials and could not be directly accounted for by movement-related activity. Finally, we did not examine sensory aspects of frontostriatal LFPs. Future work will look at other frequency bands, neuronal spike data, and at the interactions between spikes and LFPs. Because recordings in MFC and dorsomedial striatum were done in separate groups of animals, we are unable to make conclusions about the simultaneous activity of corticostriatal ensembles. In subsequent studies we hope to address these issues by more directly comparing rodent and human data, exploring changes in LFP activity during learning of temporal rules, and looking at the simultaneous activity of neuronal ensembles in both of these structures.

## Author Contributions

EE, KP, and NN designed these experiments; EE and KP collected data for these experiments, RR, EE, and NN analyzed data, and EE, RR, RK, and NN wrote the paper.

## Conflict of Interest Statement

The authors declare that the research was conducted in the absence of any commercial or financial relationships that could be construed as a potential conflict of interest.

## ABBREVIATIONS

ERP: event-related potential
LFP: local field potential
PD: Parkinson’s disease
